# The association of dietary fatty acids intake with overall and cause-specific mortality: a prospective cohort study from 1999–2018 cycles of the NHANES

**DOI:** 10.3389/fnut.2025.1468513

**Published:** 2025-02-19

**Authors:** Zhaoxiang Zhang, Lei Ding, Yali Liang, Hu Yang, Yu Zhu

**Affiliations:** ^1^Graduate School, Anhui Medical University, Hefei, China; ^2^School of Public Health, Wannan Medical College, Wuhu, China; ^3^Taizhou Central Hospital (Taizhou University Hospital), Taizhou, China

**Keywords:** mortality, saturated fatty acids, monounsaturated fatty acids, polyunsaturated fatty acids, substitution effect

## Abstract

**Background:**

Existing studies have evaluated the association of dietary fatty acids with mortality. However, the findings remained contentious. Our aimed to investigate the association of total dietary fat and each type of fatty acids with overall and cause-specific mortality.

**Methods:**

We conducted a population-based prospective cohort study derived from the U.S. NHANES from 1999 to 2018. Baseline dietary information was assessed utilizing two 24-h dietary recalls. The death status was followed up to December 31, 2019. Hazard ratio (HR) was calculated by Cox regression and competing risk model. The effects of isocaloric replacement saturated fatty acids (SFAs) with monounsaturated fatty acids (MUFAs) and polyunsaturated fatty acids (PUFAs) were estimated using the leave-one-out method.

**Results:**

A total of 49,884 U.S. adults were included. 7,347 deaths, including 2,288 and 1,652 deaths from cardio-cerebrovascular disease (CCVD) and cancer, arose during 494,277 person-years. The intake of SFAs was positively associated with an increased risk of overall mortality, with extreme-quintile HR of 1.10 (95% CI: 1.02–1.19, *P_trend_* = 0.013); whereas an inverse association of PUFAs intake with overall mortality was observed, with extreme-quintile HR of 0.87 (95% CI: 0.81–0.94, *P_trend_* = 0.001). Greater intake of PUFAs was associated with a lower risk of CCVD-and cancer-specific mortality at borderline statistical significance. The isocaloric substitutions of 5% energy from MUFAs and PUFAs for SFAs was associated with 13 and 12% lower risk of overall mortality.

**Conclusion:**

Greater intake of SFAs was positively associated with mortality, while greater intake of PUFAs was negatively associated with mortality. Reducing SFA by increasing MUFAs and PUFAs was an attractive strategy to lower mortality.

## Introduction

Dietary fat, which is one of the three macronutrients, can provide fatty acids as well as energy. Dietary fatty acids are divided into four subtypes: saturated fatty acids (SFAs), monounsaturated fatty acids (MUFAs), polyunsaturated fatty acids (PUFAs), and trans-fatty (TFAs) (1). Although the general public has paid more and more attention to the relationship between consumption of dietary fatty acids and health issues, the available evidence remains conflicting and confusing.

The controversy on the association of dietary fatty acids with health, in which the focus is the “diet-heart hypothesis,” has lasted for more than 60 years (2). Among the association of dietary fatty acids with cardiovascular disease (CVD), SFAs have been the main focus of various studies. Several prospective cohort studies have demonstrated that consuming higher SFAs was linked with an increased CVD-specific morbidity and mortality (3, 4). However, other epidemiological studies have exhibited either no association (5, 6) or even inverse association (7, 8) between SFAs consumption and CVD. Although several studies have reported that the excessive SFAs intake was shown to promote overall mortality (9, 10), these findings were refuted by others (11, 12). The inconsistency may be attributed to heterogeneity in the study populations. The UK Biobank prospective cohort study primarily included older and white participants (9), while the Prospective Urban Rural Epidemiology (PURE) study encompassed a diverse range of ethnicities across multiple countries, with a broader age range (11). A meta-analysis derived from randomized controlled trials (RCTs) have suggested that reducing SFAs intake could lower the risk of CVD but not overall mortality (13). PUFAs seem to be healthy fatty acids. A large body of evidence but not all showed that higher intake of PUFAs was associated with a lower risk of CVD morbidity and overall mortality (3, 10–12). In addition, a meta-analysis from RCTs concluded that replacing SFAs with PUFAs could reduce the risk of CVD (14). Under the context of mixed results, the Dietary Guidelines for Americans was shifted from limiting total fat to consuming less than 10% of total energy from SFAs by replacing them with unsaturated fat acids, particularly PUFAs (15). Thus, in this study, we evaluated the hypothesis that replacing SFAs with MUFAs and PUFAs could yield health benefits.

Dietary fatty acids are comprised of a variety of fatty acids, the relationship between subtypes of dietary fatty acids and health issues remain unclear. More evidence appraising the role of dietary fatty acids on health is needed to improve the dietary guidelines. In view of these circumstances, we synthetically assessed the effect of the amount and subtype of dietary fatty acids on overall and specific-cause mortality using a prospective study derived from the U.S. National Health and Nutrition Examination Survey (NHANES). Compared to the UK Biobank study (9) and the PURE study (11), participants in NHANES were younger, and the gender ratio was more balanced. Therefore, it was necessary to utilize NHANES data to examine the following two aims: (1) which subtype of fatty acids were associated with overall or cause-specific mortality? (2) what was the effect of substituting MUFAs and PUFAs for SFAs? Answering these questions could contribute to illuminating the association of dietary fatty acids with the risk of mortality.

## Methods and materials

### Study population

This cohort study was designed by longitudinally linking NHANES participants to the National Death Index (NDI) ending on December 31, 2019. The participant baseline data was available from cycles of NHANES from 1999 to 2018, which was designed to evaluate the health and nutritional level among residents of the U.S. Details of the protocol of NHANES have been described elsewhere.^1^ Concisely, it was a cross-sectional survey performed periodically every two years after 1999 by the National Center for Health Statistics (NCHS). Questionnaires and biological specimens were obtained by in person interview or mobile physical examination. The NHANES program received approval from the NCHS ethics review board, and all participants supplied informed consent (see Footnote 1).

This study included individuals aged 18 years or older, who underwent a dietary assessment during the 10 survey cycles of NHANES. Individuals were excluded according to the following criteria: (1) they had an unreliable intake of energy (less than 800 or more than 4,200 kcal/day for men; less than 600 or more than 3,500 kcal/day for women, *n* = 9,238); (2) they were not linked with mortality data (*n* = 82). After the screening, the study finally involved a cohort of 49,884 participants.

### Assessments of dietary fatty acids

In cycles of NHANES from 1999 to 2018, diet was assessed using a 24-h dietary recall in-person interview. Since 2003, participants underwent a second 24-h dietary recall via telephone conversation around 3 to10 days following the first recall. Nutrient intake was averaged among participants who underwent two dietary recalls. The nutrient density of dietary fatty acid was used instead of absolute intake to adjust for total energy intake (16). The primary exposure (total fat, total SFAs, total MUFAs and total PUFAs) was expressed as the percentage of energy, the secondary exposure (each type of dietary fatty acids) was expressed as intake per 1,000 kcal.

### Ascertainments of demographic and lifestyle factors

Demographic information (sex, age, race, education, and income) and lifestyle factors (smoking, and physical activity) were collected during household interviews at the baseline survey via standard questionnaires. Body measures and alcohol intake were collected in Mobile Examination Center. Body mass index (BMI, kg/m^2^) was determined by dividing weight by the square of height. Economic status was described as the ratio of family income to poverty. Physical activity was quantified by summing the weekly activities, and was reported in metabolic equivalent tasks (METs)-hours per week. Hypertension was characterized by individuals (1) reporting a previous diagnosis of hypertension; (2) having a systolic blood pressure equal to or greater than 140 mmHg or diastolic blood pressure equal to or greater than 90 mmHg. Diabetes was characterized by individuals (1) reporting a previous diagnosis of diabetes; (2) having a fasting glucose level equal to or greater than 7 mmol/L or a random glucose level equal to or greater than 11.1 mmol/L; (3) having a hemoglobin A1c level equal to or greater than 6.5%. Conditions of CVD, kidney failure, and cancer were identified based on individuals’ self-reporting.

### Ascertainments of deaths

The death status and cause of participants were ascertained by linking to the NDI. Mortality follow-up data was available ending on December 31, 2019. Death causes were classified following the 10th edition of International Classification of Diseases. The main endpoint was overall mortality, and secondary endpoints included mortality specifically from cardio-cerebrovascular disease (CCVD) and cancer. Duration of survival (person-years) was calculated as the time elapsed from the interview until either death or December 31, 2019, whichever occurred first. If an individual did not die until December 31, 2019, the survival time was censored.

### Statistical analysis

Quantitative variables were shown as mean with standard deviation, and discrete variables were displayed as percentages. Baseline features were compared between those with and without death using *t* tests for quantitative variables or chi-square tests for discrete variables or Wilcoxon rank-sum test for ordinal variables. The hazard ratios (HRs) and 95% confidence intervals (CIs) of overall mortality were estimated utilizing Cox regression, and HRs and 95% CIs of cause-specific mortality were estimated from competing risks model. We firstly used categorical exposures, with dietary fatty acids categorized into quartiles. The trends were estimated by a per-SD increase in each dietary fatty acid. Restricted cubic splines (RCS) was also applied with three knots in the Cox regression to flexibly model and visualize associations of dietary fatty acids with mortality (17). Multivariate models were controlled for sex, age, BMI, race, education, physical activity, economic status, drinking status, smoking status, total energy, diabetes, hypertension, kidney failure, CVD, and cancer. Given that the shape of the association of age and total energy intake with mortality retained unknown, they entered model as RCS with three knots (18).

The robustness of the findings was evaluated in sensitivity analyses. First, individuals with a history of CVD or cancer were excluded. Second, individuals who died within the initial year of follow-up were excluded. To simulate the effect of limiting total SFAs, we estimated the effects of isocaloric replacement SFAs with MUFAs and PUFAs from the multivariable nutrient density model using the leave-one-out method (19). A two-tailed *p* value <0.05 was deemed statistically significant. Statistical analyses were conducted using R software (version 4.3.0).

## Results

### Population characteristics

Of the 49,884 individuals with a mean age of 47.67 ± 19.26 years, 23,779 (47.67%) were men. Over a follow-up period of 494,277 person-years, a total of 7,347 deaths were documented, among which 2,288 were due to CCVD and 1,652 were attributed to cancer.

Baseline features of study subjects were displayed in Table 1. Briefly, individuals suffered death tended to consume less total energy, total PUFAs, MUFA 20:1 (eicosenoic acid), PUFA 18:2 (octadecadienoic acid), PUFA 18:3 (octadecatrienoic acid), PUFA 18:4 (octadecatetraenoic acid), and PUFA 22:5 (docosapentaenoic acid); but to have a higher intake of SFA 4:0 (butanoic acid), SFA 6:0 (hexanoic acid), SFA 18:0 (octadecanoic acid), MUFA 16:1 (hexadecenoic acid), MUFA 18:1 (octadecenoic acid), MUFA 22:1 (docosenoic acid), PUFA 20:5 (eicosapentaenoic acid), and PUFA 22:6 (docosahexaenoic acid). In addition, they were older, more frequently male, more frequently Non-Hispanic White, more frequently smokers, less frequently drinkers, and exhibited a lower BMI, lower level of education, lower physical activity, lower economic level, and had a higher prevalence of hypertension, diabetes, kidney failure, CVD as well as cancer. Baseline features of study subjects across NHANES cycles were exhibited in Supplementary Table S1.

**Table 1:** Baseline characteristics of study participants according to status of follow-up.^*^

Characteristics	Overall	All-cause mortality		*P*
		Alive	Death	
No. of Participants	49,884	42,537	7,347	
Age (years)	47.67 ± 19.26	43.99 ± 17.60	69.00 ± 13.79	<0.001
Male (%)	23,779 (47.67)	19,714 (46.35)	4,065 (55.33)	<0.001
BMI (%)				0.004
Underweight	900 (1.83)	745 (1.77)	155 (2.22)	
Normal	14,386 (29.29)	12,374 (29.37)	2012 (28.85)	
Overweight	16,287 (33.16)	13,803 (32.76)	2,484 (35.61)	
Obese	17,537 (35.71)	15,213 (36.11)	2,324 (33.32)	
Race (%)				<0.001
Non-Hispanic White	21,908 (43.92)	17,456 (41.04)	4,452 (60.60)	
Non-Hispanic Black	10,507 (21.06)	9,094 (21.38)	1,413 (19.23)	
Hispanic	4,018 (8.05)	3,720 (8.75)	298 (4.06)	
Other	13,451 (26.96)	12,267 (28.84)	1,184 (16.12)	
Education (%)				<0.001
Less Than High School	13,646 (27.39)	10,759 (25.31)	2,887 (39.42)	
High School Diploma	11,960 (24.00)	10,121 (23.81)	1839 (25.11)	
More Than High School	24,222 (48.61)	21,624 (50.88)	2,598 (35.47)	
Drinking status (%)				<0.001
Never	12,756 (28.45)	10,345 (27.27)	2,411 (34.92)	
Low to moderate	26,353 (58.78)	22,746 (59.97)	3,607 (52.24)	
Heavy	5,725 (12.77)	4,838 (12.76)	887 (12.85)	
Smoking status (%)				<0.001
Never	25,970 (55.06)	23,022 (57.74)	2,948 (40.42)	
Former	11,701 (24.81)	8,818 (22.11)	2,883 (39.53)	
Current	9,496 (20.13)	8,034 (20.15)	1,462 (20.05)	
Physical activity (%)				<0.001
Low level	19,896 (40.07)	15,564 (36.76)	4,332 (59.24)	
Moderate level	5,824 (11.73)	4,998 (11.81)	826 (11.29)	
High level	23,929 (48.20)	21,774 (51.43)	2,155 (29.47)	
Family income to poverty ratio (%)				<0.001
0-	14,352 (31.42)	12,044 (30.89)	2,308 (34.50)	
1.3-	17,354 (37.99)	14,378 (36.88)	2,976 (44.48)	
3.5-	13,974 (30.59)	12,568 (32.23)	1,406 (21.02)	
Diabetes (%)	7,419 (14.88)	5,262 (12.38)	2,157 (29.39)	<0.001
Hypertension (%)	18,160 (37.15)	13,121 (31.49)	5,039 (69.79)	<0.001
Failing kidney (%)	1,465 (3.16)	925 (2.37)	540 (7.42)	<0.001
CVD (%)	5,191 (11.23)	2,850 (7.31)	2,341 (32.42)	<0.001
Cancer (%)	4,412 (9.51)	2,825 (7.22)	1,587 (21.75)	<0.001
Total energy (kcal/day)	2001.04 ± 727.64	2038.99 ± 732.93	1781.34 ± 654.37	<0.001
Total fat (%)	33.41 ± 8.00	33.42 ± 7.97	33.33 ± 8.17	0.342
Total SFAs (%)	10.80 ± 3.38	10.79 ± 3.36	10.86 ± 3.51	0.145
Total MUFAs (%)	12.07 ± 3.41	12.06 ± 3.39	12.14 ± 3.51	0.051
Total PUFAs (%)	7.48 ± 2.90	7.52 ± 2.90	7.24 ± 2.91	<0.001
SFA 4:0 (butanoic acid, g/day)	0.23 ± 0.17	0.23 ± 0.17	0.24 ± 0.19	0.007
SFA 6:0 (hexanoic acid, g/day)	0.13 ± 0.10	0.13 ± 0.10	0.14 ± 0.11	0.024
SFA 8:0 (octanoic acid, g/day)	0.11 ± 0.09	0.11 ± 0.09	0.11 ± 0.11	0.918
SFA 10:0 (decanoic acid, g/day)	0.21 ± 0.14	0.21 ± 0.14	0.20 ± 0.15	0.076
SFA 12:0 (dodecanoic acid, g/day)	0.36 ± 0.45	0.36 ± 0.45	0.35 ± 0.45	0.597
SFA 14:0 (tetradecanoic acid, g/day)	0.98 ± 0.56	0.99 ± 0.55	0.98 ± 0.59	0.761
SFA 16:0 (hexadecanoic acid, g/day)	6.58 ± 1.86	6.58 ± 1.84	6.59 ± 1.92	0.687
SFA 18:0 (octadecanoic acid, g/day)	3.01 ± 0.97	2.99 ± 0.96	3.10 ± 1.02	<0.001
MUFA 16:1 (hexadecenoic acid, g/day)	0.57 ± 0.27	0.57 ± 0.27	0.59 ± 0.29	<0.001
MUFA 18:1 (octadecenoic acid, g/day)	12.47 ± 3.59	12.45 ± 3.57	12.58 ± 3.69	0.009
MUFA 20:1 (eicosenoic acid, g/day)	0.12 ± 0.10	0.13 ± 0.10	0.11 ± 0.11	<0.001
MUFA 22:1 (docosenoic acid, mg/day)	16.76 ± 64.71	16.34 ± 63.63	19.18 ± 70.55	0.001
PUFA 18:2 (octadecadienoic acid, g/day)	7.32 ± 2.91	7.36 ± 2.90	7.07 ± 2.90	<0.001
PUFA 18:3 (octadecatrienoic acid, g/day)	0.76 ± 0.38	0.76 ± 0.39	0.73 ± 0.38	<0.001
PUFA 18:4 (octadecatetraenoic acid, mg/day)	5.17 ± 14.60	5.26 ± 14.20	4.66 ± 16.72	0.004
PUFA 20:4 (eicosatetraenoic acid, mg/day)	73.68 ± 52.08	73.72 ± 51.15	73.47 ± 57.17	0.725
PUFA 20:5 (eicosapentaenoic acid, mg/day)	19.26 ± 55.79	18.82 ± 53.25	21.85 ± 68.63	<0.001
PUFA 22:5 (docosapentaenoic acid, mg/day)	10.81 ± 17.58	10.96 ± 17.09	9.96 ± 20.13	<0.001
PUFA 22:6 (docosahexaenoic acid, mg/day)	38.63 ± 86.20	38.01 ± 83.68	42.27 ± 99.48	0.001

^*^Values were expressed as mean ± SD or percentage. BMI, body mass index; CVD, cardiovascular diseases; MUFAs, monounsaturated fatty acids; PUFAs, polyunsaturated fatty acids; SFAs, saturated fatty acids.

### Dietary fatty acids and overall mortality

**Figure 1:**
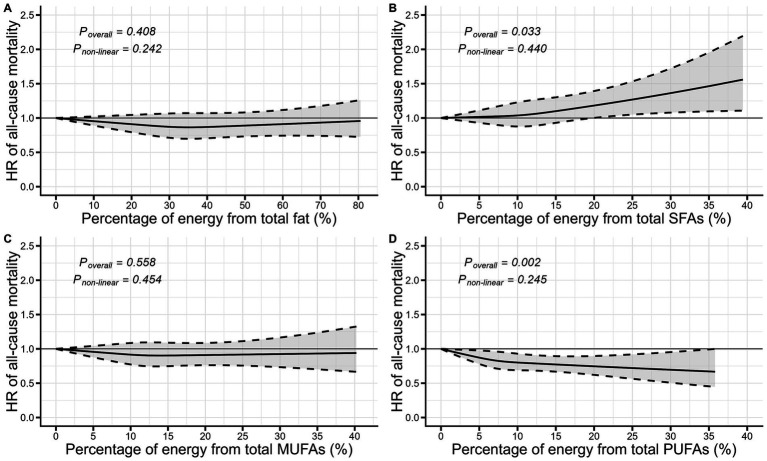
Visualization of the dose–response relationship between dietary fatty acids and all-cause mortality based on restricted cubic splines. ^**^The model was adjusted for sex, age, BMI, race, education, physical activity, family income to poverty ratio, drinking status, smoking status, energy, hypertension, diabetes, failing kidney, CVD, and cancer. BMI, Body mass index; CVD, Cardiovascular disease; HR, Hazard ratio; MUFAs, monounsaturated fatty acids; PUFAs, polyunsaturated fatty acids; SFAs, saturated fatty acids.

After multivariable adjustment, the total SFAs intake was suggested to have a modest but significant association with an increased risk of overall mortality. As shown in Figure 1, the lowest risk of death was observed among individuals who approximately ingested less than 10% of total energy from SFAs (*P_overall_* = 0.033 based on RCS model). Furthermore, the multivariable-adjusted HR for individuals comparing the highest quartile to the lowest quartile was 1.10 (95% CI: 1.02–1.19, *P_trend_* = 0.013, Table 2). Consuming higher amounts of PUFAs was linked to a reduced risk of overall mortality (*P_overall_* = 0.002 based on RCS model; Figure 1). Compared to the lowest quartile of total PUFAs intake, the multivariable-adjusted HR of overall mortality for highest quartile were 0.87 (95% CI: 0.81–0.94, *P_trend_* = 0.001, Table 2). However, there was no significant association of total fat and MUFAs with overall mortality (Figure 1; Table 2).

**Table 2:** The HRs and 95% CIs of all-cause mortality according to quartiles of dietary fatty acids intake.*

Dietary fatty acids	Quartiles				Per 1-SD	*P_trend_*
	Quartile 1	Quartile 2	Quartile 3	Quartile 4		
Total fat^a^	1	1.00 (0.94–1.07)	1.06 (0.99–1.13)	1.14 (1.07–1.22)	1.06 (1.03–1.09)	<0.001
Total fat^b^	1	0.97 (0.90–1.04)	0.98 (0.92–1.06)	1.00 (0.93–1.08)	0.99 (0.96–1.02)	0.517
Total SFAs^a^	1	1.04 (0.98–1.11)	1.02 (0.96–1.09)	1.13 (1.06–1.20)	1.06 (1.03–1.09)	<0.001
Total SFAs^b^	1	1.02 (0.94–1.09)	1.05 (0.97–1.13)	1.10 (1.02–1.19)	1.04 (1.01–1.08)	0.013
Total MUFAs^a^	1	1.01 (0.95–1.08)	1.00 (0.93–1.06)	1.04 (0.98–1.11)	1.02 (0.99–1.05)	0.211
Total MUFAs^b^	1	1.00 (0.93–1.08)	0.99 (0.92–1.07)	0.99 (0.92–1.06)	0.99 (0.96–1.02)	0.439
Total PUFAs^a^	1	0.98 (0.92–1.05)	1.05 (0.99–1.12)	1.06 (0.99–1.13)	1.03 (1.00–1.06)	0.050
Total PUFAs^b^	1	0.92 (0.86–0.99)	0.93 (0.87–1.00)	0.87 (0.81–0.94)	0.95 (0.92–0.98)	0.001
SFA 4:0 (butanoic acid)^a^	1	0.87 (0.81–0.93)	0.84 (0.79–0.90)	0.97 (0.91–1.03)	1.02 (0.99–1.05)	0.229
SFA 4:0 (butanoic acid)^b^	1	0.99 (0.92–1.06)	1.02 (0.95–1.10)	1.08 (1.00–1.16)	1.04 (1.00–1.07)	0.024
SFA 6:0 (hexanoic acid)^a^	1	0.87 (0.81–0.93)	0.87 (0.81–0.92)	1.09 (1.03–1.16)	1.08 (1.06–1.11)	<0.001
SFA 6:0 (hexanoic acid)^b^	1	0.97 (0.90–1.04)	1.04 (0.97–1.12)	1.08 (1.00–1.15)	1.04 (1.01–1.08)	0.004
SFA 8:0 (octanoic acid)^a^	1	0.89 (0.83–0.95)	0.86 (0.81–0.92)	1.06 (1.00–1.13)	1.06 (1.03–1.08)	<0.001
SFA 8:0 (octanoic acid)^b^	1	1.02 (0.95–1.10)	1.00 (0.93–1.08)	1.07 (1.00–1.15)	1.02 (0.99–1.04)	0.164
SFA 10:0 (decanoic acid)^a^	1	0.91 (0.85–0.97)	0.89 (0.84–0.95)	1.09 (1.02–1.16)	1.08 (1.05–1.11)	<0.001
SFA 10:0 (decanoic acid)^b^	1	1.04 (0.96–1.11)	1.05 (0.97–1.13)	1.07 (1.00–1.15)	1.04 (1.01–1.07)	0.016
SFA 12:0 (dodecanoic acid)^a^	1	0.91 (0.85–0.97)	0.97 (0.91–1.03)	1.09 (1.02–1.16)	1.03 (1.02–1.04)	<0.001
SFA 12:0 (dodecanoic acid)^b^	1	1.03 (0.96–1.11)	1.01 (0.94–1.09)	1.02 (0.95–1.10)	0.99 (0.98–1.01)	0.476
SFA 14:0 (tetradecanoic acid)^a^	1	0.93 (0.87–0.99)	0.89 (0.83–0.95)	0.98 (0.92–1.05)	1.02 (0.99–1.05)	0.234
SFA 14:0 (tetradecanoic acid)^b^	1	1.03 (0.95–1.10)	1.05 (0.98–1.13)	1.07 (0.99–1.15)	1.04 (1.00–1.07)	0.024
SFA 16:0 (hexadecanoic acid)^a^	1	1.02 (0.95–1.08)	1.00 (0.94–1.07)	1.11 (1.04–1.18)	1.05 (1.02–1.08)	0.001
SFA 16:0 (hexadecanoic acid)^b^	1	0.99 (0.92–1.06)	1.01 (0.94–1.09)	1.08 (1.01–1.17)	1.04 (1.01–1.08)	0.019
SFA 18:0 (octadecanoic acid)^a^	1	1.04 (0.97–1.11)	1.10 (1.03–1.18)	1.20 (1.13–1.28)	1.10 (1.07–1.14)	<0.001
SFA 18:0 (octadecanoic acid)^b^	1	0.99 (0.92–1.07)	1.07 (0.99–1.16)	1.10 (1.02–1.19)	1.05 (1.02–1.09)	0.002
MUFA 16:1 (hexadecenoic acid)^a^	1	0.93 (0.87–0.99)	0.89 (0.84–0.95)	0.90 (0.85–0.96)	0.98 (0.96–1.01)	0.213
MUFA 16:1 (hexadecenoic acid)^b^	1	0.95 (0.88–1.02)	1.00 (0.93–1.08)	0.99 (0.92–1.07)	1.01 (0.98–1.04)	0.520
MUFA 18:1 (octadecenoic acid)^a^	1	1.01 (0.95–1.08)	0.99 (0.93–1.06)	1.06 (1.00–1.13)	1.03 (1.00–1.05)	0.080
MUFA 18:1 (octadecenoic acid)^b^	1	1.00 (0.93–1.08)	1.00 (0.93–1.08)	0.98 (0.91–1.06)	0.99 (0.96–1.02)	0.463
MUFA 20:1 (eicosenoic acid)^a^	1	0.89 (0.84–0.95)	0.89 (0.83–0.95)	0.95 (0.89–1.01)	1.00 (0.98–1.02)	0.649
MUFA 20:1 (eicosenoic acid)^b^	1	0.94 (0.88–1.01)	0.92 (0.85–0.99)	0.90 (0.83–0.97)	0.96 (0.93–0.98)	0.001
MUFA 22:1 (docosenoic acid)^a^	1	0.98 (0.92–1.05)	1.03 (0.97–1.10)	1.04 (0.98–1.10)	1.00 (1.00–1.01)	<0.001
MUFA 22:1 (docosenoic acid)^b^	1	0.98 (0.91–1.06)	0.97 (0.90–1.04)	0.92 (0.86–0.99)	1.00 (0.99–1.00)	0.102
PUFA 18:2 (octadecadienoic acid)^a^	1	1.00 (0.94–1.06)	1.04 (0.97–1.11)	1.03 (0.97–1.10)	1.02 (0.99–1.05)	0.221
PUFA 18:2 (octadecadienoic acid)^b^	1	0.91 (0.85–0.98)	0.94 (0.88–1.01)	0.87 (0.81–0.94)	0.95 (0.92–0.98)	0.001
PUFA 18:3 (octadecatrienoic acid)^a^	1	1.05 (0.99–1.12)	1.19 (1.12–1.27)	1.19 (1.11–1.27)	1.06 (1.04–1.08)	<0.001
PUFA 18:3 (octadecatrienoic acid)^b^	1	0.92 (0.85–0.98)	0.95 (0.88–1.02)	0.85 (0.79–0.92)	0.94 (0.92–0.97)	<0.001
PUFA 18:4 (octadecatetraenoic acid)^a^	1	0.75 (0.65–0.86)	0.82 (0.77–0.88)	0.82 (0.77–0.87)	1.00 (1.00–1.00)	0.510
PUFA 18:4 (octadecatetraenoic acid)^b^	1	0.96 (0.82–1.12)	0.97 (0.90–1.04)	0.97 (0.91–1.04)	1.00 (1.00–1.00)	0.829
PUFA 20:4 (eicosatetraenoic acid)^a^	1	0.87 (0.81–0.93)	0.88 (0.83–0.94)	1.05 (0.99–1.12)	1.06 (1.03–1.08)	<0.001
PUFA 20:4 (eicosatetraenoic acid)^b^	1	0.93 (0.87–1.00)	0.96 (0.89–1.03)	0.97 (0.90–1.04)	0.99 (0.96–1.02)	0.477
PUFA 20:5 (eicosapentaenoic acid)^a^	1	0.94 (0.88–1.00)	0.85 (0.80–0.91)	0.95 (0.90–1.01)	1.00 (1.00–1.01)	0.001
PUFA 20:5 (eicosapentaenoic acid)^b^	1	0.94 (0.88–1.01)	0.93 (0.86–1.00)	0.92 (0.86–0.99)	1.00 (1.00–1.00)	0.504
PUFA 22:5 (docosapentaenoic acid)^a^	1	0.81 (0.76–0.86)	0.75 (0.70–0.80)	0.85 (0.80–0.91)	1.00 (0.99–1.02)	0.649
PUFA 22:5 (docosapentaenoic acid)^b^	1	0.94 (0.88–1.01)	0.91 (0.85–0.99)	0.95 (0.88–1.01)	0.99 (0.97–1.00)	0.115
PUFA 22:6 (docosahexaenoic acid)^a^	1	0.95 (0.89–1.02)	0.99 (0.93–1.06)	1.04 (0.98–1.11)	1.02 (1.01–1.02)	<0.001
PUFA 22:6 (docosahexaenoic acid)^b^	1	0.97 (0.90–1.04)	0.96 (0.89–1.03)	0.91 (0.85–0.98)	1.00 (0.99–1.00)	0.303

^a^The model was not adjusted for any covariates. ^b^The model was adjusted for sex, age, BMI, race, education, physical activity, family income to poverty ratio, drinking status, smoking status, energy, hypertension, diabetes, failing kidney, CVD, and cancer. ^*^BMI, body mass index; CI, confidence interval; CVD: cardiovascular disease; HR, hazard ratio; MUFAs: monounsaturated fatty acids; PUFAs: polyunsaturated fatty acids; SFAs: saturated fatty acids.

Among each type of fatty acids (Table 2), the multivariable-adjusted HRs of overall mortality comparing the highest quartile to the lowest quartile of SFA 4:0 (butyric acid), SFA 6:0 (caproic acid), SFA 8:0 (caprylic acid), SFA 10:0 (capric acid), SFA 16:0 (palmitic acid) and SFA 18:0 (stearic acid) were 1.08 (95% CI: 1.00–1.16), 1.08 (95% CI: 1.00–1.15), 1.07 (95% CI: 1.00–1.15), 1.07 (95% CI: 1.00–1.15), 1.08 (95% CI: 1.01–1.17) and 1.10 (95% CI: 1.02–1.19), respectively. However, contrasted with the lowest quartile, the highest quartile of MUFA 20:1 (eicosenoic acid) and MUFA 22:1 (docosenoic acid) could reduce a 10% (HR = 0.90, 95% CI: 0.83–0.97) and 8% (HR = 0.92, 95% CI: 0.86–0.99) risk of overall mortality. Likewise, for PUFAs subtypes, the highest quartile of PUFA 18:2 (linoleic acid), PUFA 18:3 (linolenic acid), PUFA 20:5 (eicosapentaenoic acid), and PUFA 22:6 (docosahexaenoic acid) experienced a reduced risk of overall mortality, with extreme-quintile HRs of 0.87 (95% CI: 0.81–0.94), 0.85 (95% CI: 0.79–0.92), 0.92 (95% CI: 0.86–0.99) and 0.91 (95% CI: 0.85–0.98). The positive association between butyric acid, caproic acid, palmitic acid, and stearic acid with overall mortality was confirmed by the RCS model. Similarly, the negative association of linoleic acid and linolenic acid with overall mortality was also confirmed by the RCS model (Supplementary Figure S1).

### Dietary fatty acids and cause-specific mortality

The association between dietary fatty acids intake and mortality specifically related to CCVD and cancer, estimated from competing risks model, was presented in Table 3. We identified an association between total PUFAs and CCVD-specific mortality at borderline statistical significance. In contrast to the lowest quartile, the multivariable-adjusted HRs were 0.89 (95% CI: 0.78–1.01) for the second quartile, 0.83 (95% CI: 0.73–0.95) for the third quartile, 0.89 (95% CI: 0.78–1.01) for the fourth quartile. For individual subtypes of PUFAs, higher PUFA 18:2 (linoleic acid) and PUFA 18:3 (linolenic acid), PUFA 18:4 (octadecatetraenoic acid), PUFA 20:4 (eicosatetraenoic acid), PUFA 20:5 (eicosapentaenoic acid), PUFA 22:5 (docosapentaenoic acid), and PUFA 22:6 (docosahexaenoic acid) intake might reduce the risk of CCVD-specific mortality by 4 to 21%. No significant associations of total fat, total SFAs and MUFAs with CCVD-specific mortality was found. Similarly, total PUFAs was associated with cancer-specific mortality at borderline statistical significance, with a multivariable-adjusted HRs of 0.88 (95% CI: 0.76–1.02) for the second quartile, 0.85 (95% CI: 0.73–0.99) for the third quartile, and 0.90 (95% CI: 0.78–1.05) for the fourth quartile. Higher PUFA 18:2 (linoleic acid), PUFA 18:3 (linolenic acid) and PUFA 18:4 (octadecatetraenoic acid) intake might reduce the risk of cancer-specific mortality by 5 to 27%.

**Table 3:** Adjusted HRs and 95% CIs of cause-specific mortality according to quartiles of dietary fatty acids intake^*^.

Dietary fat acids	CCVD-specific mortality				Cancer-specific mortality			
	Quartile 1	Quartile 2	Quartile 3	Quartile 4	Quartile 1	Quartile 2	Quartile 3	Quartile 4
Total fat	1	0.91 (0.79–1.04)	0.95 (0.83–1.09)	0.96 (0.84–1.10)	1	0.86 (0.74–1.01)	0.91 (0.78–1.06)	1.01 (0.87–1.18)
Total SFAs	1	0.97 (0.85–1.11)	0.94 (0.81–1.08)	1.08 (0.94–1.23)	1	0.90 (0.77–1.05)	0.95 (0.82–1.11)	1.00 (0.86–1.16)
Total MUFAs	1	1.00 (0.88–1.15)	1.03 (0.90–1.18)	0.98 (0.85–1.12)	1	0.95 (0.81–1.11)	0.94 (0.81–1.10)	1.03 (0.88–1.20)
Total PUFAs	1	0.89 (0.78–1.01)	0.83 (0.73–0.95)	0.89 (0.78–1.01)	1	0.88 (0.76–1.02)	0.85 (0.73–0.99)	0.90 (0.78–1.05)
SFA 4:0 (butanoic acid)	1	0.91 (0.80–1.04)	0.94 (0.82–1.07)	0.93 (0.81–1.06)	1	0.94 (0.81–1.10)	0.90 (0.77–1.05)	1.03 (0.89–1.20)
SFA 6:0 (hexanoic acid)	1	0.92 (0.81–1.05)	0.96 (0.84–1.10)	0.92 (0.80–1.04)	1	0.93 (0.80–1.08)	0.88 (0.76–1.03)	1.02 (0.88–1.18)
SFA 8:0 (octanoic acid)	1	0.97 (0.85–1.11)	0.95 (0.83–1.08)	0.99 (0.87–1.12)	1	0.94 (0.81–1.09)	0.92 (0.79–1.08)	1.03 (0.88–1.19)
SFA 10:0 (decanoic acid)	1	1.03 (0.90–1.17)	0.95 (0.83–1.09)	0.99 (0.87–1.12)	1	0.98 (0.84–1.14)	0.99 (0.85–1.15)	0.96 (0.82–1.12)
SFA 12:0 (dodecanoic acid)	1	0.91 (0.79–1.03)	0.90 (0.79–1.03)	0.96 (0.84–1.09)	1	0.93 (0.80–1.09)	0.98 (0.84–1.14)	0.96 (0.83–1.12)
SFA 14:0 (tetradecanoic acid)	1	1.01 (0.89–1.15)	0.99 (0.86–1.13)	1.03 (0.90–1.17)	1	1.06 (0.92–1.24)	1.07 (0.92–1.25)	1.03 (0.89–1.21)
SFA 16:0 (hexadecanoic acid)	1	0.96 (0.84–1.09)	0.98 (0.85–1.12)	1.04 (0.91–1.19)	1	0.92 (0.79–1.07)	0.96 (0.82–1.12)	1.02 (0.88–1.19)
SFA 18:0 (octadecanoic acid)	1	0.95 (0.83–1.09)	1.01 (0.88–1.16)	1.04 (0.91–1.19)	1	0.92 (0.79–1.08)	1.02 (0.87–1.19)	1.06 (0.90–1.23)
MUFA 16:1 (hexadecenoic acid)	1	0.97 (0.84–1.11)	0.98 (0.86–1.12)	1.05 (0.92–1.20)	1	1.03 (0.88–1.20)	1.07 (0.91–1.25)	1.13 (0.97–1.31)
MUFA 18:1 (octadecenoic acid)	1	1.05 (0.92–1.21)	1.06 (0.93–1.22)	0.99 (0.87–1.14)	1	0.88 (0.75–1.03)	0.93 (0.80–1.09)	0.98 (0.84–1.14)
MUFA 20:1 (eicosenoic acid)	1	0.97 (0.86–1.10)	0.86 (0.75–0.98)	0.88 (0.77–1.00)	1	0.80 (0.70–0.93)	0.92 (0.79–1.06)	0.75 (0.64–0.88)
MUFA 22:1 (docosenoic acid)	1	0.93 (0.82–1.07)	0.89 (0.78–1.02)	0.99 (0.88–1.13)	1	0.88 (0.75–1.02)	0.93 (0.80–1.08)	0.91 (0.79–1.05)
PUFA 18:2 (octadecadienoic acid)	1	0.87 (0.77–0.99)	0.84 (0.74–0.96)	0.88 (0.77–1.00)	1	0.87 (0.75–1.01)	0.82 (0.71–0.96)	0.89 (0.76–1.04)
PUFA 18:3 (octadecatrienoic acid)	1	0.93 (0.82–1.06)	0.89 (0.78–1.02)	0.89 (0.77–1.01)	1	0.83 (0.72–0.97)	0.83 (0.71–0.96)	0.84 (0.72–0.98)
PUFA 18:4 (octadecatetraenoic acid)	1	0.79 (0.59–1.06)	0.82 (0.72–0.94)	0.92 (0.82–1.04)	1	0.73 (0.53–1.01)	0.76 (0.65–0.89)	0.95 (0.83–1.09)
PUFA 20:4 (eicosatetraenoic acid)	1	0.81 (0.71–0.93)	0.90 (0.79–1.02)	0.87 (0.76–0.99)	1	1.03 (0.89–1.20)	1.03 (0.88–1.20)	1.13 (0.97–1.32)
PUFA 20:5 (eicosapentaenoic acid)	1	0.86 (0.76–0.98)	0.82 (0.72–0.94)	0.90 (0.79–1.01)	1	0.88 (0.75–1.03)	0.92 (0.79–1.07)	1.02 (0.89–1.18)
PUFA 22:5 (docosapentaenoic acid)	1	0.83 (0.73–0.95)	0.86 (0.76–0.99)	0.96 (0.85–1.09)	1	0.95 (0.82–1.10)	0.89 (0.76–1.04)	0.98 (0.84–1.13)
PUFA 22:6 (docosahexaenoic acid)	1	0.90 (0.78–1.03)	0.91 (0.80–1.04)	0.93 (0.82–1.06)	1	1.03 (0.88–1.21)	1.10 (0.94–1.28)	1.10 (0.94–1.28)

^*^The model was adjusted for sex, age, BMI, race, education, physical activity, family income to poverty ratio, drinking status, smoking status, energy, hypertension, diabetes, failing kidney, CVD, and cancer.

When excluding participants with a history of comorbidities or those who died within the first year of follow-up, we found that the association was robust and remained similar (Supplementary Table S2).

### Assessing the isocaloric substitution effect of MUFAs and PUFAs for SFAs

Isocalorically replacing 5% energy from SFAs with equivalent amount from MUFAs was linked to a 13% (HR = 0.87, 95% CI: 0.80–0.94, *p* < 0.001) lower risk of overall mortality. The isocaloric substitution of 5% energy from MUFAs for SFAs was not significantly linked to CCVD-specific mortality (HR = 0.94, 95% CI: 0.81–1.09, *p* = 0.419) and cancer-specific mortality (HR = 0.90, 95% CI: 0.76–1.07, *p* = 0.234). Similarly, isocalorically replacing 5% energy from SFAs with equivalent amount from PUFAs was linked to a 12% (HR = 0.88, 95% CI: 0.83–0.94, *p* < 0.001) lower risk of overall mortality. The isocaloric substitution of 5% energy from PUFAs for SFAs was not significantly linked to CCVD-specific mortality (HR = 0.95, 95% CI: 0.85–1.06, *p* = 0.333) and cancer-specific mortality (HR = 0.93, 95% CI: 0.82–1.06, *p* = 0.274).

## Discussion

In this study, we revealed that increased consumption of total SFAs was linked to a slight elevation in the risk of overall mortality, whereas increased intake of total PUFAs was linked to a reduction in risk of overall mortality. In addition, when the participant limited total SFAs intake by replacing them with total MUFAs and PUFAs, the risk of overall mortality might decrease.

Previous research has investigated the link between consumption of dietary fat and mortality (9, 11, 20), and the finding highlighted the complexity and diversity effect of dietary fat on health. Although a cohort study derived from UK Biobank (9) included 195,658 participants reported a non-significant relationship between total fat and overall mortality, a U-shaped tendency was shown in the penalized cubic splines. A U-shaped pattern with the lowest risk of death at 30–40% energy from total fat was reproduced in Korea National Health and Nutrition Examination Survey (20) of 42,192 participants. Our finding echoed this pattern, indicating that total fat exhibited a U-shaped relationship with overall mortality, albeit lacking statistical significance. However, the PURE study (11) conducted in 18 countries from five continents suggested that reducing total fat intake could be detrimental with no safety threshold for overall mortality. Regarding the association between total fat and mortality specifically related to CCVD and cancer, our finding aligned with the results presented by a meta-analysis of prospective cohort studies (21), which also found no significant association between total fat intake and mortality related to CVD and cancer.

When examining the health effects of dietary fat, it is important to consider the components of dietary fat. We observed that total SFAs consumption was mildly associated with an elevated risk of overall mortality. This finding corroborated UK Biobank study (9) that revealed a significant positive association between consumption of total SFAs and overall mortality. However, the PURE study (11) found an opposite result that consumption of total SFAs showed a negative correlation with overall mortality. Therefore, the finding from PURE study did not endorse the dietary guidelines advocating for limiting total SFAs to less than 10% of energy. The divergent findings across studies are likely not only attributed to the inconsistency in demographic characteristics such as age and sex among the study populations, but also to variations in the energy from carbohydrates. Most participants from countries with low to moderate income levels in the PURE study consumed more carbohydrate than those in UK Biobank study, with the estimated proportion of energy intake from carbohydrate decreasing from 61 to 50%. A cohort study showed a U-shape pattern between carbohydrate consumption and mortality, indicating that both reduced and elevated consumption of carbohydrate were linked to a higher risk of mortality compared to moderate carbohydrate consumption (22). Given that higher carbohydrate intake was linked to an increased risk of mortality (20, 22), the available evidence suggested that substituting carbohydrate with fat or protein might reduce risk of mortality (11, 22). Consuming more carbohydrate could compress the fat intake, resulting in a relatively narrower range of fat intake. The inverse relationship between total SFAs and overall mortality in PURE study was an artifact caused by left truncation, concealing a U-shaped pattern.

We did not identify a significant correlation between MUFAs and overall mortality, while ingesting total PUFAs was linked to a reduced risk of overall mortality. Previous cohort studies reported a similar association between intake of total PUFAs and the risk of overall mortality (3, 10–12, 23). Additionally, several cohort studies have confirmed that higher plasma PUFAs levels were also linked to a reduced risk of overall mortality (24, 25). In this study, we explored the association between each type of PUFAs and mortality. Both the quartile Cox model and the RCS Cox model indicated a negative association of linoleic acid and linolenic acid with overall mortality. Furthermore, we found that total PUFAs might be linked to a reduced risk of CCVD-and cancer-specific mortality, consistent with results from cohort studies and meta-analyses (21, 23). Although the underlying molecular mechanisms are still unclear, lipid metabolism and inflammatory might been implicated. On the one hand, abnormal lipid metabolism links dietary fat to the onset of multiple chronic diseases. Linolenic acid, the precursor of the omega-3 fatty acids, and linoleic acid, the precursor of the omega-6 fatty acids, are essential fatty acids. Linolenic acid is widely recognized for possessing the capability to lower plasma concentrations of triglycerides (TG) and low-density lipoproteins cholesterol (LDL-C) (26). Several studies have shown that SFAs were responsible for increasing plasma low-density lipoproteins cholesterol (LDL-C) concentrations, and replacing SFAs with MUFAs and PUFAs might lead to a decrease in TC and LDL-C concentration, reducing the risk of CVD events and CVD-related deaths (27–29). Although the relationship between linoleic acid and lipid metabolism remains unclear, meta-analyses showed that linoleic acid reduced plasma cholesterol (30) and lowed the risk of CVD events and related deaths (31). On the other hand, omega-3 fatty acids have anti-inflammatory effects via suppressing nuclear factor kappa B (NFκB) and stimulating peroxisome proliferator-activated receptors (PPARs) (32). This anti-inflammatory effect of omega-3 fatty acids might contribute to lowering death via reducing inflammation-driven chronic diseases including CVD and cancer (21). Despite the potential for increased inflammation with higher omega-6 fatty acids intake, existing research does not support a direct link between inflammation and omega-6 fatty acids consumption (29).

Therefore, a substitution analysis was carried out to mimic the impact of altering dietary components. We found that the isocaloric substitutions of total MUFAs and PUFAs for total SFAs was linked to a reduced risk of overall mortality. Potential benefits of replacing SFA with MUFAs and PUFAs have been reported in some cohort studies (12, 23). Collectively, these findings indicated that increasing consumption of MUFAs-or PUFAs-rich foods yield a bounty of health benefits.

The research faced numerous limitations. First, the potential effect of reverse causation on the findings could not be discounted, as individuals with chronic illnesses might alter their dietary patterns. However, the findings were substantially consistent after excluding participants who either had pre-existing comorbidities or died within the initial year of follow-up. Second, dietary information was collected using the 24-h dietary recall method at baseline. While this method may introduce recall bias and may not fully capture long-term dietary patterns, it remains a cost-effective and low-burden tool for gathering dietary data in nutritional epidemiology. Third, despite controlling for several covariates, residual confounding from unmeasured factors could still influence the interpretation of the results. Fourth, the relatively small number of deaths from CCVD and cancer during the 9.91 years of average follow-up limited the statistical power of the study.

In conclude, consuming higher SFAs was linked to a greater risk of overall mortality. Conversely, a higher intake of PUFAs was linked to a reduced risk of overall mortality. Furthermore, replacing SFAs with MUFAs and PUFAs resulted in a substantial decrease in the risk of overall mortality. To translate these findings into practical recommendations, public health strategies should encourage individuals to replace foods high in SFAs with those rich in MUFAs and PUFAs, particularly omega-3 fatty acids. Such dietary modifications could be an effective approach to improving long-term health outcomes at the population level.

## Data Availability

The datasets presented in this study can be found in online repositories. The names of the repository/repositories and accession number(s) can be found at: https://wwwn.cdc.gov/nchs/nhanes/Default.aspx.

## Footnotes

^1^ https://www.cdc.gov/nchs/nhanes/about_nhanes.htm
