# Supplementary material for: The association of dietary fatty acids intake with overall and cause-specific mortality: a prospective cohort study from 1999–2018 cycles of the NHANES
Source: Front Nutr. 2025 Feb 19;12:1468513. doi: 10.3389/fnut.2025.1468513 (PMC11879808; doi:10.3389/fnut.2025.1468513)
Supplement: Supplementary file 1 [file Data_Sheet_1.docx]

**Table S1 Baseline characteristics of study participants according to the cycle of NHANES^*^**

| **Characteristics** | **Overall** | **NHANES cycle** | | | | | | | | | |
| --- | --- | --- | --- | --- | --- | --- | --- | --- | --- | --- | --- |
|  |  | **1999-2000** | **2001-2002** | **2003-2004** | **2005-2006** | **2007-2008** | **2009-2010** | **2011-2012** | **2013-2014** | **2015-2016** | **2017-2018** |
| No. of Participants | 49884 | 4378 | 4865 | 4749 | 4847 | 5430 | 5813 | 4872 | 5121 | 5068 | 4741 |
| Age (years) | 47.7±19.3 | 47.1±20.2 | 46.4±20.0 | 47.6±21.0 | 45.2±20.0 | 49.3±18.7 | 48.2±18.6 | 47.2±18.6 | 47.1±18.4 | 48.3±18.5 | 49.8±18.5 |
| Male (%) | 23779(47.7) | 2005(45.8) | 2277(46.8) | 2265(47.7) | 2299(47.4) | 2644(48.7) | 2778(47.8) | 2407(49.4) | 2416(47.2) | 2423(47.8) | 2265(47.8) |
| BMI (%) |  |  |  |  |  |  |  |  |  |  |  |
| Underweight | 900(1.8) | 77(1.8) | 101(2.2) | 90(1.9) | 87(1.8) | 93(1.7) | 99(1.7) | 103(2.1) | 93(1.8) | 73(1.5) | 84(1.8) |
| Normal | 14386(29.3) | 1424(32.9) | 1496(32.3) | 1487(31.9) | 1466(30.7) | 1486(27.8) | 1577(27.4) | 1471(30.6) | 1483(29.2) | 1331(26.5) | 1165(24.9) |
| Overweight | 16287(33.2) | 1476(34.1) | 1671(36.1) | 1581(33.9) | 1594(33.4) | 1842(34.4) | 1912(33.2) | 1515(31.5) | 1619(31.9) | 1592(31.7) | 1485(31.7) |
| Obese | 17537(35.7) | 1354(31.3) | 1365(29.5) | 1509(32.3) | 1632(34.1) | 1928(36.0) | 2171(37.7) | 1718(35.7) | 1884(37.1) | 2023(40.3) | 1953(41.7) |
| Race (%) |  |  |  |  |  |  |  |  |  |  |  |
| Non-Hispanic white | 21908(43.9) | 1867(42.6) | 2475(50.9) | 2453(51.7) | 2321(47.9) | 2539(46.8) | 2784(47.9) | 1834(37.6) | 2228(43.5) | 1703(33.6) | 1704(35.9) |
| Non-Hispanic black | 10507(21.1) | 820(18.7) | 928(19.1) | 980(20.6) | 1149(23.7) | 1126(20.7) | 1036(17.8) | 1304(26.8) | 1008(19.7) | 1065(21.0) | 1091(23.0) |
| Hispanic | 4018(8.1) | 283(6.5) | 204(4.2) | 143(3.0) | 148(3.1) | 604(11.1) | 580(10.0) | 481(9.9) | 467(9.1) | 676(13.3) | 432(9.1) |
| Other | 13451(27.0) | 1408(32.2) | 1258(25.9) | 1173(24.7) | 1229(25.4) | 1161(21.4) | 1413(24.3) | 1253(25.7) | 1418(27.7) | 1624(32.0) | 1514(31.9) |
| Education (%) |  |  |  |  |  |  |  |  |  |  |  |
| Less Than High School | 13646(27.4) | 1713(39.2) | 1520(31.3) | 1446(30.5) | 1361(28.1) | 1682(31.0) | 1658(28.6) | 1140(23.4) | 1072(20.9) | 1166(23.0) | 888(18.8) |
| High School Diploma | 11960(24.0) | 1026(23.5) | 1170(24.1) | 1200(25.3) | 1234(25.5) | 1347(24.8) | 1360(23.4) | 1040(21.4) | 1207(23.6) | 1172(23.1) | 1204(25.4) |
| More Than High School | 24222(48.6) | 1626(37.3) | 2170(44.7) | 2097(44.2) | 2249(46.4) | 2398(44.2) | 2782(48.0) | 2689(55.2) | 2840(55.5) | 2728(53.8) | 2643(55.8) |
| Drinking status (%) |  |  |  |  |  |  |  |  |  |  |  |
| Never | 12756(28.5) | 1282(34.3) | 1348(32.1) | 1273(31.6) | 1271(31.1) | 1449(29.7) | 1352(26.7) | 1250(27.5) | 1427(29.2) | 1598(33.1) | 506(11.0) |
| Low to moderate | 26353(58.8) | 2002(53.5) | 2320(55.3) | 2275(56.5) | 2255(55.2) | 2789(57.2) | 2926(57.8) | 2658(58.5) | 2864(58.6) | 2706(56.1) | 3558(77.6) |
| Heavy | 5725(12.8) | 458(12.2) | 525(12.5) | 482(12.0) | 559(13.7) | 636(13.0) | 788(15.6) | 635(14.0) | 598(12.2) | 522(10.8) | 522(11.4) |
| Smoking status (%) |  |  |  |  |  |  |  |  |  |  |  |
| Never | 25970(55.1) | 2092(53.3) | 2272(51.6) | 2136(50.3) | 2299(53.0) | 2748(53.1) | 3002(54.1) | 2603(56.5) | 3001(58.6) | 3005(59.4) | 2812(59.3) |
| Former | 11701(24.8) | 1056(26.9) | 1177(26.7) | 1174(27.6) | 1117(25.7) | 1319(25.5) | 1372(24.7) | 1080(23.4) | 1142(22.3) | 1140(22.5) | 1124(23.7) |
| Current | 9496(20.1) | 780(19.9) | 952(21.6) | 939(22.1) | 923(21.3) | 1110(21.4) | 1170(21.1) | 925(20.1) | 977(19.1) | 915(18.1) | 805(17.0) |
| Physical activity (%) |  |  |  |  |  |  |  |  |  |  |  |
| Low level | 19896(40.1) | 2044(46.9) | 2113(43.6) | 1961(41.5) | 1823(37.7) | 2239(41.5) | 2359(40.7) | 1817(37.5) | 1921(37.7) | 1909(38.0) | 1710(36.3) |
| Moderate level | 5824(11.7) | 587(13.5) | 667(13.8) | 733(15.5) | 711(14.7) | 504(9.3) | 621(10.7) | 533(11.0) | 552(10.8) | 472(9.4) | 444(9.4) |
| High level | 23929(48.2) | 1731(39.7) | 2067(42.6) | 2036(43.0) | 2299(47.6) | 2656(49.2) | 2820(48.6) | 2501(51.6) | 2629(51.5) | 2639(52.6) | 2551(54.2) |
| Family income to poverty ratio (%) |  |  |  |  |  |  |  |  |  |  |  |
| 0- | 14352(31.4) | 1185(31.6) | 1278(28.1) | 1381(30.7) | 1292(27.9) | 1517(30.7) | 1780(33.7) | 1620(36.0) | 1641(34.5) | 1452(31.6) | 1206(28.7) |
| 1.3- | 17354(38.0) | 1418(37.9) | 1758(38.7) | 1763(39.2) | 1788(38.6) | 1913(38.7) | 1987(37.6) | 1541(34.2) | 1628(34.3) | 1845(40.2) | 1713(40.8) |
| 3.5- | 13974(30.6) | 1142(30.5) | 1507(33.2) | 1352(30.1) | 1551(33.5) | 1514(30.6) | 1511(28.6) | 1345(29.8) | 1484(31.2) | 1292(28.2) | 1276(30.4) |
| Diabetes (%) | 7419(14.9) | 520(11.9) | 567(11.7) | 586(12.3) | 534(11.0) | 908(16.7) | 899(15.5) | 781(16.0) | 778(15.2) | 914(18.0) | 932(19.7) |
| Hypertension (%) | 18160(37.1) | 1512(35.1) | 1572(33.0) | 1696(36.9) | 1536(32.5) | 2112(39.8) | 2145(37.7) | 1803(37.7) | 1822(36.1) | 1951(38.9) | 2011(43.3) |
| Failing kidney (%) | 1465(3.2) | 120(3.1) | 107(2.4) | 122(2.9) | 110(2.5) | 156(3.0) | 140(2.5) | 166(3.6) | 149(3.1) | 208(4.3) | 187(4.2) |
| CVD (%) | 5191(11.2) | 426(10.9) | 469(10.7) | 583(13.7) | 463(10.7) | 618(12.0) | 591(10.7) | 479(10.4) | 482(10.0) | 531(11.0) | 549(12.3) |
| Cancer (%) | 4412(9.5) | 315(8.0) | 405(9.2) | 408(9.6) | 357(8.2) | 515(10.0) | 566(10.2) | 405(8.8) | 466(9.7) | 488(10.1) | 487(10.8) |
| Total energy (kcal/day) | 2001.0±727.6 | 1986.7±767.6 | 2042.9±769.7 | 2041.6±734.5 | 2052.5±731.7 | 1950.6±720.5 | 1990.2±711.1 | 2017.1±724.7 | 1991.6±701.9 | 1969.7±699.8 | 1976.3±714.6 |
| Total fat (%) | 33.4±8.0 | 32.2±9.2 | 32.9±9.2 | 33.2±7.5 | 33.2±7.6 | 33.1±7.8 | 32.5±7.6 | 32.8±7.4 | 33.9±7.5 | 34.6±7.8 | 35.9±7.9 |
| Total SFAs (%) | 10.8±3.4 | 10.6±3.8 | 10.4±3.8 | 10.8±3.2 | 11.0±3.3 | 10.8±3.2 | 10.6±3.3 | 10.4±3.2 | 10.8±3.2 | 11.1±3.4 | 11.5±3.4 |
| Total MFAs (%) | 12.1±3.4 | 12.1±4.0 | 12.1±3.9 | 12.4±3.2 | 12.2±3.2 | 12.2±3.4 | 11.7±3.2 | 11.7±3.2 | 11.8±3.3 | 12.2±3.4 | 12.3±3.3 |
| Total PFAs (%) | 7.5±2.9 | 6.8±3.3 | 6.8±3.2 | 7.1±2.6 | 7.1±2.6 | 7.2±2.6 | 7.3±2.6 | 7.9±2.8 | 8.0±2.8 | 8.1±2.9 | 8.5±3.0 |
| SFA 4:0 (g/day) | 0.2±0.2 | 0.2±0.2 | 0.2±0.2 | 0.2±0.2 | 0.3±0.2 | 0.2±0.2 | 0.2±0.2 | 0.2±0.2 | 0.2±0.2 | 0.2±0.2 | 0.2±0.1 |
| SFA 6:0 (g/day) | 0.1±0.1 | 0.1±0.1 | 0.1±0.1 | 0.1±0.1 | 0.1±0.1 | 0.1±0.1 | 0.1±0.1 | 0.1±0.1 | 0.1±0.1 | 0.1±0.1 | 0.1±0.1 |
| SFA 8:0 (g/day) | 0.1±0.1 | 0.1±0.1 | 0.1±0.1 | 0.1±0.1 | 0.1±0.1 | 0.1±0.1 | 0.1±0.1 | 0.1±0.1 | 0.1±0.1 | 0.1±0.1 | 0.1±0.1 |
| SFA 10:0 (g/day) | 0.2±0.1 | 0.2±0.1 | 0.2±0.2 | 0.2±0.1 | 0.2±0.1 | 0.2±0.1 | 0.2±0.1 | 0.2±0.1 | 0.2±0.1 | 0.2±0.2 | 0.2±0.2 |
| SFA 12:0 (g/day) | 0.4±0.5 | 0.3±0.4 | 0.3±0.4 | 0.3±0.4 | 0.3±0.4 | 0.3±0.4 | 0.3±0.4 | 0.3±0.4 | 0.4±0.5 | 0.4±0.5 | 0.4±0.6 |
| SFA 14:0 (g/day) | 1.0±0.6 | 1.0±0.6 | 1.0±0.6 | 1.0±0.5 | 1.0±0.5 | 1.0±0.5 | 1.0±0.5 | 0.9±0.5 | 1.0±0.5 | 1.0±0.6 | 1.0±0.6 |
| SFA 16:0 (g/day) | 6.6±1.9 | 6.6±2.2 | 6.4±2.0 | 6.6±1.7 | 6.6±1.7 | 6.5±1.7 | 6.4±1.8 | 6.3±1.7 | 6.6±1.8 | 6.8±1.8 | 7.0±1.9 |
| SFA 18:0 (g/day) | 3.0±1.0 | 3.0±1.1 | 3.0±1.1 | 3.1±0.9 | 3.2±0.9 | 3.1±0.9 | 3.0±0.9 | 2.9±0.9 | 2.9±0.9 | 2.9±0.9 | 3.0±1.0 |
| MFA 16:1 (g/day) | 0.6±0.3 | 0.7±0.4 | 0.6±0.3 | 0.6±0.3 | 0.6±0.3 | 0.6±0.3 | 0.5±0.2 | 0.5±0.2 | 0.5±0.2 | 0.6±0.3 | 0.6±0.3 |
| MFA 18:1 (g/day) | 12.5±3.6 | 12.5±4.2 | 12.5±4.1 | 12.8±3.3 | 12.6±3.4 | 12.7±3.5 | 12.1±3.4 | 12.1±3.4 | 11.9±3.4 | 12.6±3.5 | 12.9±3.5 |
| MFA 20:1 (g/day) | 0.1±0.1 | 0.1±0.1 | 0.1±0.2 | 0.1±0.1 | 0.1±0.1 | 0.1±0.1 | 0.1±0.1 | 0.1±0.1 | 0.2±0.1 | 0.1±0.1 | 0.2±0.1 |
| MFA 22:1 (mg/day) | 16.8±64.7 | 19.8±59.1 | 20.2±134.8 | 18.7±46.9 | 16.9±48.9 | 18.0±59.3 | 13.0±54.7 | 14.7±48.2 | 15.2±47.1 | 15.4±43.1 | 16.8±55.2 |
| PFA 18:2 (g/day) | 7.3±2.9 | 6.7±3.3 | 6.7±3.2 | 6.9±2.6 | 7.0±2.7 | 7.0±2.7 | 7.1±2.7 | 7.7±2.8 | 7.8±2.8 | 7.9±2.9 | 8.4±3.0 |
| PFA 18:3 (g/day) | 0.8±0.4 | 0.7±0.4 | 0.7±0.4 | 0.7±0.3 | 0.7±0.3 | 0.7±0.3 | 0.7±0.3 | 0.8±0.4 | 0.8±0.4 | 0.9±0.4 | 0.9±0.4 |
| PFA 18:4 (mg/day) | 5.2±14.6 | 2.3±14.0 | 2.7±14.5 | 3.9±11.2 | 6.4±15.5 | 8.4±17.8 | 5.7±13.2 | 5.7±13.7 | 5.0±13.7 | 5.9±15.8 | 4.7±14.4 |
| PFA 20:4 (mg/day) | 73.7±52.1 | 70.9±61.8 | 64.8±55.9 | 68.3±46.0 | 70.3±48.1 | 72.2±49.0 | 75.1±52.5 | 73.9±49.6 | 79.8±51.0 | 80.1±50.8 | 80.5±53.4 |
| PFA 20:5 (mg/day) | 19.3±55.8 | 18.7±64.3 | 20.5±74.1 | 20.4±52.4 | 23.3±64.0 | 23.3±66.8 | 17.4±42.4 | 17.4±45.4 | 17.3±45.0 | 16.9±46.3 | 17.3±49.0 |
| PFA 22:5 (mg/day) | 10.8±17.6 | 8.4±19.7 | 8.4±21.1 | 8.8±15.2 | 9.7±17.3 | 10.0±18.2 | 11.8±14.0 | 12.3±17.5 | 12.8±17.5 | 12.2±15.6 | 13.1±18.3 |
| PFA 22:6 (mg/day) | 38.6±86.2 | 37.9±89.3 | 38.0±101.5 | 40.0±78.1 | 42.3±87.4 | 43.7±95.2 | 36.6±69.6 | 36.6±82.7 | 37.9±86.3 | 36.4±80.8 | 36.8±88.8 |

*Values were mean±SD or percentages.

Abbreviations: BMI: body mass index; CVD: cardiovascular disease; MFAs: monounsaturated fatty acids; PFA: polyunsaturated fatty acids; SFAs: saturated fatty acids.

Abbreviations: BMI, body mass index; CCVD: cardio-cerebrovascular diseases; CI, confidence interval; CVD: cardiovascular diseases; HR, hazard ratio; MUFAs: monounsaturated fatty acids; PUFAs: polyunsaturated fatty acids; SFAs: saturated fatty acids.

**Table S2 The HRs and 95% CIs of mortality from all-cause, CCVD and cancer estimated from sensitivity analyses^*^**

| **Dietary fat acids** | **All-Cause Mortality** | | |  | **CCVD-specific mortality** | | |  | **Cancer-specific mortality** | | |
| --- | --- | --- | --- | --- | --- | --- | --- | --- | --- | --- | --- |
|  | **Quartile2** | **Quartile3** | **Quartile4** |  | **Quartile2** | **Quartile3** | **Quartile4** |  | **Quartile2** | **Quartile3** | **Quartile4** |
| Total fat | 0.96(0.86-1.06) | 0.96(0.87-1.06) | 1.00(0.90-1.10) |  | 0.97(0.80-1.17) | 0.91(0.75-1.11) | 1.06(0.88-1.28) |  | 0.94(0.76-1.16) | 1.01(0.81-1.24) | 1.05(0.85-1.29) |
| Total SFAs | 0.97(0.88-1.08) | 1.05(0.95-1.17) | 1.07(0.96-1.18) |  | 0.90(0.74-1.09) | 0.97(0.79-1.18) | 1.09(0.90-1.32) |  | 1.00(0.82-1.24) | 0.96(0.78-1.20) | 1.04(0.85-1.29) |
| Total MFAs | 0.95(0.86-1.06) | 0.95(0.86-1.05) | 0.97(0.88-1.08) |  | 0.96(0.79-1.16) | 0.98(0.81-1.19) | 1.00(0.83-1.21) |  | 0.86(0.69-1.07) | 0.92(0.74-1.14) | 1.07(0.87-1.31) |
| Total PFAs | 0.92(0.84-1.01) | 0.87(0.79-0.96) | 0.86(0.77-0.95) |  | 0.92(0.77-1.10) | 0.81(0.67-0.98) | 0.92(0.76-1.12) |  | 0.96(0.78-1.17) | 0.96(0.78-1.17) | 0.91(0.74-1.13) |
| SFA 4:0 | 0.98(0.89-1.09) | 1.08(0.97-1.20) | 1.08(0.98-1.19) |  | 0.92(0.76-1.11) | 0.91(0.75-1.11) | 0.94(0.78-1.13) |  | 1.09(0.88-1.34) | 0.99(0.80-1.23) | 1.11(0.90-1.37) |
| SFA 6:0 | 0.97(0.87-1.07) | 1.10(0.99-1.21) | 1.08(0.98-1.19) |  | 0.91(0.75-1.10) | 0.94(0.78-1.15) | 0.95(0.79-1.14) |  | 1.02(0.83-1.25) | 0.99(0.80-1.23) | 1.06(0.86-1.30) |
| SFA 8:0 | 1.01(0.91-1.11) | 1.02(0.92-1.13) | 1.07(0.97-1.19) |  | 1.04(0.86-1.25) | 0.98(0.80-1.19) | 1.04(0.87-1.25) |  | 0.97(0.79-1.19) | 0.88(0.70-1.09) | 1.08(0.88-1.32) |
| SFA 10:0 | 1.06(0.96-1.17) | 1.09(0.99-1.21) | 1.09(0.98-1.20) |  | 1.11(0.92-1.33) | 0.92(0.76-1.13) | 1.02(0.84-1.23) |  | 1.07(0.87-1.32) | 1.12(0.91-1.38) | 1.01(0.81-1.25) |
| SFA 12:0 | 1.03(0.94-1.14) | 1.06(0.96-1.17) | 1.02(0.92-1.13) |  | 0.94(0.78-1.14) | 0.97(0.80-1.18) | 0.95(0.79-1.15) |  | 1.02(0.83-1.25) | 0.99(0.80-1.22) | 0.97(0.79-1.19) |
| SFA 14:0 | 1.01(0.91-1.12) | 1.07(0.96-1.18) | 1.07(0.97-1.19) |  | 1.03(0.86-1.25) | 0.97(0.80-1.18) | 1.02(0.84-1.23) |  | 1.20(0.97-1.48) | 1.19(0.96-1.47) | 1.13(0.91-1.40) |
| SFA 16:0 | 0.96(0.87-1.06) | 0.97(0.88-1.08) | 1.07(0.97-1.18) |  | 0.97(0.80-1.18) | 1.02(0.84-1.25) | 1.13(0.93-1.37) |  | 0.90 (0.73-1.12) | 1.01(0.82-1.25) | 1.04(0.84-1.28) |
| SFA 18:0 | 1.01(0.91-1.12) | 1.02(0.92-1.13) | 1.08(0.98-1.20) |  | 0.96(0.78-1.17) | 1.01(0.83-1.23) | 1.08(0.89-1.32) |  | 1.00(0.80-1.25) | 1.14(0.92-1.42) | 1.11(0.89-1.38) |
| MFA 16:1 | 1.00(0.90-1.12) | 0.99(0.89-1.10) | 1.03(0.93-1.13) |  | 1.08(0.88-1.32) | 1.03(0.85-1.26) | 1.14(0.94-1.38) |  | 0.93(0.74-1.16) | 1.04(0.84-1.30) | 1.22(0.99-1.50) |
| MFA 18:1 | 0.97(0.88-1.07) | 0.95(0.86-1.05) | 0.98(0.89-1.09) |  | 1.00(0.82-1.21) | 1.03(0.85-1.25) | 1.00(0.82-1.21) |  | 0.81(0.65-1.01) | 0.86(0.69-1.06) | 1.04(0.85-1.28) |
| MFA 20:1 | 0.99(0.90-1.09) | 0.96(0.87-1.06) | 0.90(0.81-1.00) |  | 1.11(0.93-1.32) | 0.84(0.69-1.03) | 1.02(0.84-1.23) |  | 0.86(0.70-1.04) | 1.00(0.82-1.21) | 0.75(0.60-0.93) |
| MFA 22:1 | 1.02(0.92-1.13) | 0.98(0.89-1.09) | 0.94(0.85-1.04) |  | 0.97(0.80-1.17) | 0.89(0.74-1.08) | 1.01(0.84-1.21) |  | 0.98(0.79-1.20) | 0.91(0.74-1.12) | 0.97(0.79-1.18) |
| PFA 18:2 | 0.92(0.84-1.02) | 0.89(0.80-0.98) | 0.86(0.78-0.96) |  | 0.89(0.74-1.07) | 0.83(0.68-1.00) | 0.90(0.75-1.10) |  | 0.94(0.77-1.15) | 0.88(0.72-1.08) | 0.87(0.70-1.08) |
| PFA 18:3 | 0.94(0.85-1.04) | 0.90(0.82-1.00) | 0.86(0.78-0.96) |  | 0.87(0.72-1.04) | 0.86(0.71-1.03) | 0.91(0.75-1.10) |  | 0.92(0.76-1.13) | 0.91(0.74-1.12) | 0.88(0.71-1.09) |
| PFA 18:4 | 0.99(0.79-1.25) | 1.02(0.92-1.13) | 0.94(0.86-1.03) |  | 1.09(0.71-1.67) | 0.99(0.81-1.20) | 0.93(0.79-1.11) |  | 0.76(0.46-1.24) | 0.79(0.63-0.99) | 1.06(0.88-1.27) |
| PFA 20:4 | 0.94(0.85-1.04) | 0.93(0.84-1.03) | 0.92(0.84-1.02) |  | 0.86(0.71-1.04) | 0.86(0.71-1.04) | 0.97(0.81-1.16) |  | 1.16(0.94-1.43) | 1.00(0.81-1.25) | 1.16(0.94-1.43) |
| PFA 20:5 | 0.96(0.86-1.06) | 0.91(0.82-1.00) | 0.89(0.81-0.98) |  | 0.83(0.69-1.00) | 0.83(0.69-1.01) | 0.81(0.68-0.97) |  | 0.96(0.77-1.19) | 0.96(0.77-1.19) | 1.13(0.93-1.37) |
| PFA 22:5 | 0.97(0.88-1.07) | 0.90(0.81-1.01) | 0.87(0.79-0.96) |  | 0.79(0.66-0.96) | 0.88(0.73-1.07) | 0.97(0.81-1.15) |  | 1.11(0.92-1.35) | 0.90(0.72-1.13) | 1.00(0.81-1.22) |
| PFA 22:6 | 0.96(0.87-1.06) | 0.95(0.86-1.05) | 0.85(0.77-0.94) |  | 0.93(0.77-1.13) | 0.93(0.77-1.12) | 0.90(0.74-1.08) |  | 1.16(0.93-1.45) | 1.15(0.93-1.42) | 1.13(0.91-1.40) |

^*^ The model was adjusted for sex, age, BMI, race, education, physical activity, family income to poverty ratio, drinking status, smoking status, energy, hypertension, diabetes, failing kidney, CVD, and cancer. Quartile1 was set as reference.

Abbreviations: BMI, body mass index; CCVD: cardio-cerebrovascular diseases; CI, confidence interval; CVD: cardiovascular diseases; HR, hazard ratio; MUFAs: monounsaturated fatty acids; PUFAs: polyunsaturated fatty acids; SFAs: saturated fatty acids.


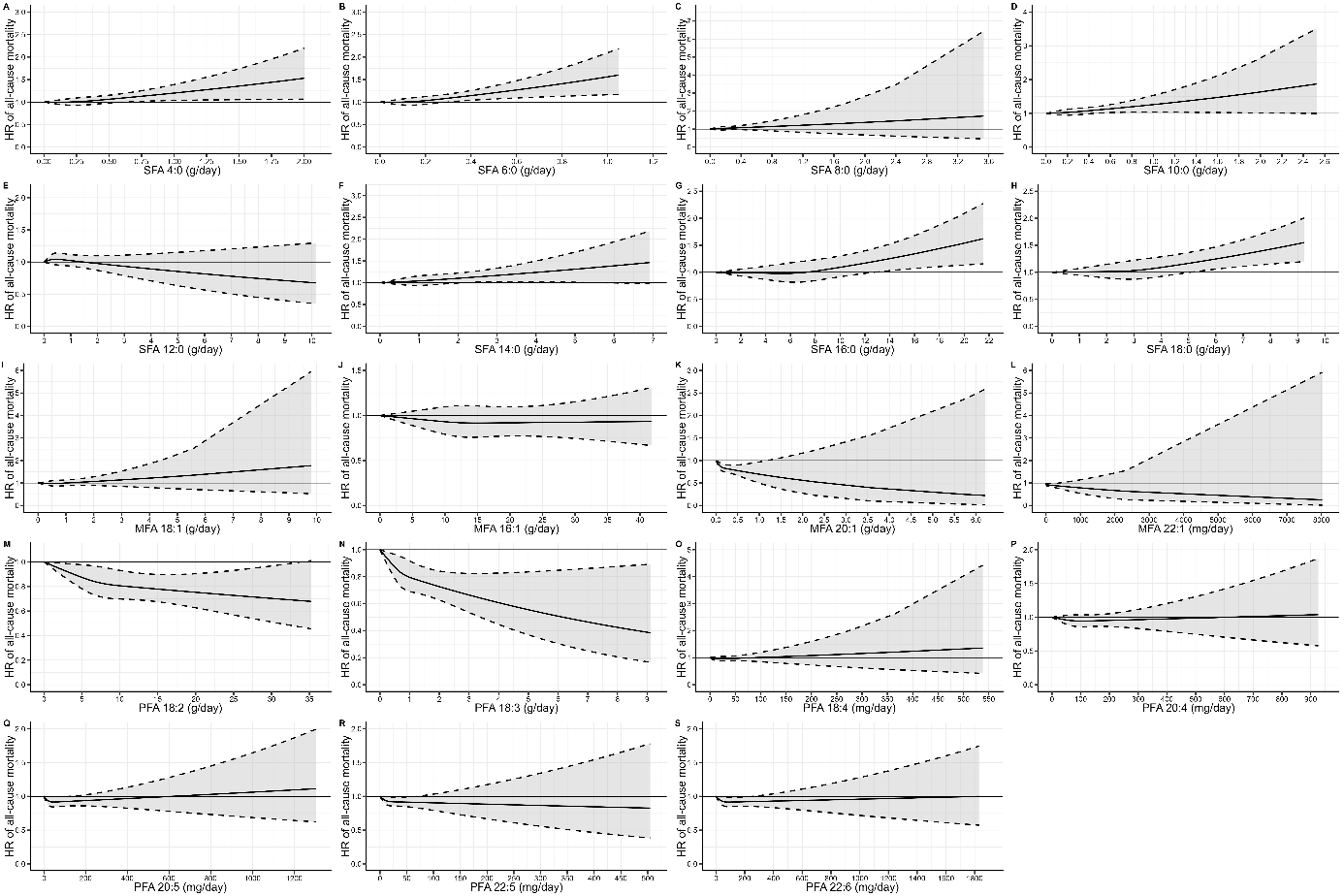


**Figure S1 Visualization of the dose-response relationship** **between each type of dietary fatty acids and all-cause mortality based on restricted cubic splines^*^**

**^*^** The model was adjusted for sex, age, BMI, race, education, physical activity, family income to poverty ratio, drinking status, smoking status, energy, hypertension, diabetes, failing kidney, CVD, and cancer.

Abbreviations: BMI, Body mass index; CVD: Cardiovascular disease; HR, Hazard ratio; MUFAs: monounsaturated fatty acids; PUFAs: polyunsaturated fatty acids; SFAs: saturated fatty acids.
